# Metabolomic Characterization of Ovarian Epithelial Carcinomas by HRMAS-NMR Spectroscopy

**DOI:** 10.1155/2011/174019

**Published:** 2011-04-26

**Authors:** D. Ben Sellem, K. Elbayed, A. Neuville, F.-M. Moussallieh, G. Lang-Averous, M. Piotto, J.-P. Bellocq, I. J. Namer

**Affiliations:** ^1^Biophysics and Nuclear Medicine Department, University Hospitals of Strasbourg, 67098 Strasbourg, France; ^2^University of Strasbourg, CNRS LINC UMR 7237, 67081 Strasbourg, France; ^3^University of Strasbourg, Institute of Chemistry, CNRS UMR 7177, 67081 Strasbourg, France; ^4^Pathology Department, University Hospitals of Strasbourg, 67098, France; ^5^Bruker BioSpin, 67160 Wissembourg, France

## Abstract

*Objectives*. The objectives of the present study are to determine if a metabolomic study by HRMAS-NMR can (i) discriminate between different histological types of epithelial ovarian carcinomas and healthy ovarian tissue, (ii) generate statistical models capable of classifying borderline tumors and (iii) establish a potential relationship with patient's survival or response to chemotherapy. *Methods*. 36 human epithelial ovarian tumor biopsies and 3 healthy ovarian tissues were studied using ^1^H HRMAS NMR spectroscopy and multivariate statistical analysis. *Results*. The results presented in this study demonstrate that the three histological types of epithelial ovarian carcinomas present an effective metabolic pattern difference. Furthermore, a metabolic signature specific of serous (N-acetyl-aspartate) and mucinous (N-acetyl-lysine) carcinomas was found. The statistical models generated in this study are able to predict borderline tumors characterized by an intermediate metabolic pattern similar to the normal ovarian tissue. Finally and importantly, the statistical model of serous carcinomas provided good predictions of both patient's survival rates and the patient's response to chemotherapy. *Conclusions*. Despite the small number of samples used in this study, the results indicate that metabolomic analysis of intact tissues by HRMAS-NMR is a promising technique which might be applicable to the therapeutic management of patients.

## 1. Introduction

Epithelial ovarian carcinoma is one of the most frequent malignancies of the female genital tract and represents the leading cause of death among women with gynecologic cancer. The overall 5-year survival still remains at about 44% [1]. Clinicians face two key problems: late diagnosis at advanced stages which is more difficult to treat and resistance to treatment of the majority of recurrences. Thus, there is a need for improved imaging methods to predict treatment response and detect tumor recurrence not invasively [2, 3]. The unfavorable statistics in ovarian cancer patients reflect, in part, a poor understanding of the molecular pathogenesis of this heterogeneous malignancy [4]. 

Several studies have been performed for the metabolic characterization of ovarian carcinomas using *ex vivo* ^1^H nuclear magnetic resonance (NMR) spectroscopy in tumor tissues [5, 6], cyst fluid [7–9], or cell lines [10] and by *in vivo* ^1^H MR spectroscopy [9, 11–14]. So far, no studies have yet been performed using ^1^H high-resolution magic angle spinning (HRMAS) NMR spectroscopy. This technique affords a detailed and accurate analysis of the metabolic composition of intact tissue specimens without resorting to time-consuming extraction techniques. 

The objectives of the present study were to determine if a metabolomics study using ^1^H-HRMAS NMR spectroscopy could (i) discriminate between different histological types of epithelial ovarian carcinoma and healthy ovarian tissue, (ii) generate statistical models capable of classifying borderline tumors, and (iii) establish a potential relationship with patient's survival or response to chemotherapy. 

## 2. Materials and Methods

### 2.1. Patients Population

Thirty six human epithelial ovarian tumor biopsies obtained from the tumor bank of the University Hospitals of Strasbourg were selected retrospectively for this preliminary study according to the following criteria: (1) absence of mixed epithelial and nonepithelial carcinoma, (2) absence of any anticancer treatment prior to surgery, (3) tumor tissue sample quantitatively and qualitatively (viable tumor/necrosis ratio) adequate to perform a correct HRMAS analysis, (4) tissue specimens collected immediately after surgery and stored at –80°C, and (5) absence of tissue samples pollution by the histopathological fixing medium. 

Histopathological examination of the selected specimens revealed 22 serous, 4 endometrioid, and 5 mucinous carcinoma as well as 5 borderline tumors (3 serous and 2 mucinous). The tumors were also graded using the FIGO [15] and the Silverberg [16] grading systems. Normal contralateral ovarian tissue from three of these patients served as controls.

A clinical longitudinal study including response to the first-line chemotherapy, the date of recurrence, and the survival was performed on 15 patients with serous ovarian cancer. 

### 2.2. HRMAS Analysis

HRMAS spectra were recorded on a Bruker Avance III 500 spectrometer operating at a proton frequency of 500.13 MHz following an established protocol [17]. This instrument is installed at the Hautepierre University Hospital in Strasbourg and is dedicated to the analysis of biopsies by HRMAS. It is operated by qualified scientific and medical personnel in the context of the CARMeN project which aims at the creation of an extensive metabolic database covering most current human tumors. The amount of tumoral tissue used ranged from 16 to 20 mg. All NMR experiments were conducted at a temperature of 4°C on samples spinning at 3502 Hz. For each biopsy sample, a one-dimensional proton spectrum using a Carr-Purcell-Meiboom-Gill (CPMG) pulse sequence was acquired (Bruker *cpmgpr1d* pulse sequence). The number of loops was set to 328 resulting in a the CPMG pulse train of total length 93 ms. The CPMG experiment was acquired with the following parameters: sweep width 14.2 ppm, number of points 32 k, relaxation delay 2 s, and acquisition time 2.3 s. A total of 128 FID were acquired resulting in an acquisition time of 10 min. The FID was multiplied by an exponential weighing function corresponding to a line broadening of 0.3 Hz prior to Fourier transformation. All spectra were processed using automatic base-line correction routines. ^1^H spectra were referenced by setting the lactate doublet chemical shift to 1.33 ppm. In order to compare the metabolic content present in different samples and to obtain absolute concentration values, a synthetic digital Eretic signal was added to all the 1D spectra at 10.5 ppm [18, 19]. The principles used for the measurement of the concentrations of metabolites in biopsy specimens are similar to those used in liquid samples except that the results are expressed in mmole·kg^−1^.

For the purpose of confirming resonance assignments, complementary 2D homonuclear ^1^H-^1^H TOCSY and heteronuclear ^1^H-^13^C HSQC experiments were recorded on 8 samples [17]: 3 serous carcinoma, 1 endometrioid carcinoma, 1 mucinous carcinoma and 3 healthy ovarian tissues, respectively. 

### 2.3. Statistical Analysis

Principal component analysis (PCA) and partial least square discriminant analysis (PLS-DA) were conducted on 1D ^1^H CPMG HRMAS spectra of the healthy ovarian tissues and of the serous, endometrioid, and mucinous carcinomas using the same established protocol [17]. The spectral region between 4.7 and 0.5 ppm of each 1D CPMG NMR spectrum was automatically binned into regions of 0.01 ppm using the AMIX software (Bruker GmbH, Germany). This procedure minimizes the effect of peak shifts due to pH variations. The peak integral within each 0.01 ppm region was computed and normalized with respect to the total integral of the spectrum in the 4.7–0.5 ppm region. This process generated an *X* data matrix containing 421 columns (chemical shifts) and 36 rows (corresponding to healthy ovarian tissues and serous, endometrioid and mucinous carcinomas). Data sets were then imported into the SIMCA P 11.0 software (Umetrics AB, Umeå, Sweden) and preprocessed using unit variance scaling of the *X* columns by weighing each integral region by 1/SD_k_, where SD_k_ represents the standard deviation of the *k*th column in the *X* matrix. The *X* matrix was analyzed using principal component analysis (PCA) within the SIMCA P 11.0 software package. In a PCA analysis, the first component (PC1) represents the axis that explains the largest variance in the set of samples studied. The subsequent components (PC2, PC3,…) explain the residual variance in decreasing order of importance. Each axis is built using a linear combination of the different signals in the spectra (chemical shifts). This procedure allows to evaluate quickly the quality of the data and to identify possible outliers. After the PCA analysis, partial least square discriminant analysis (PLS-DA) [20] was conducted in order to build a statistical model that optimizes the separation between the two classes of patients. The number of components of the PLS-DA model was determined by cross-validation. The first PLS-DA component is the one that is best correlated with the *Y* response of the samples. The *Y* response contains the information used to build the model (histology, patient's survival, and response to chemotherapy). Each component is built using a linear combination of the different signals in the spectra. The class membership of each sample was iteratively predicted, using the results to generate a goodness of fit measure (*Q* ^2^ = 1− PRESS/SS) for the overall model. PRESS is the predicted squared sum of error and represents the squared differences between observed and predicted *Y* values when each sample is kept out of model development and SS is the residual sum of squares of the previous dimension. The maximum theoretical value for *Q* ^2^ is equal to 1 for a perfect prediction. However, a *Q* ^2^ value superior to 0.5 is generally considered to be a decent predictor. 

## 3. Results

**Figure 1:**
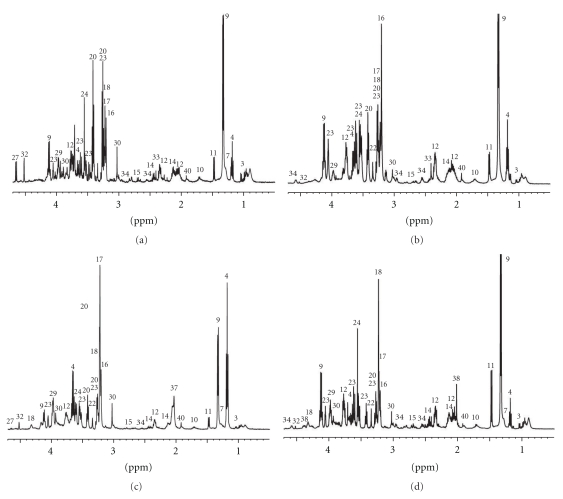
Representative 1D ^1^H CPMG HRMAS spectra of healthy ovarian tissues (a) and endometrial (b), mucinous (c) and serous (d) carcinomas. Partial metabolite assignment in the 4.7–0.5 ppm region is indicated. The numbers refer to the metabolites listed in Table 1. The metabolic content of healthy and cancerous biopsies can be directly compared, since the intensity of each spectrum was normalized with respect to the amplitude of the digital Eretic signal and the weight of biopsy present in each sample.

**Figure 2:**
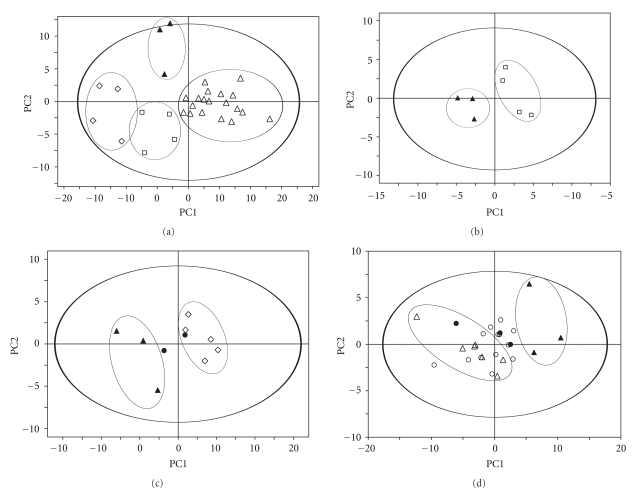
Score plot of the first two principal components (PC1, PC2) from PLS-DA model obtained when comparing: (a) Healthy ovarian tissues (filled triangle) versus the 3 epithelial carcinomas: mucinous (open diamond), endometrioid (open square) and serous (open triangle). Model parameters: *R* ^2^ *Y* = 0.75, *Q* ^2^ = 0.50. (b) Healthy ovarian tissues (full triangle) versus endometrioid carcinomas (open square). Model parameters: *R* ^2^ *Y* = 0.96, *Q* ^2^ = 0.45. (c) Healthy ovarian tissues (full triangle) versus mucinous carcinomas (open diamond). Model parameters: *R* ^2^ *Y* = 0.94, *Q* ^2^ = 0.69. (d) Healthy ovarian tissues (full triangle) versus high Silverberg score (grade III) of serous carcinomas (open triangle). Model parameters: *R* ^2^ *Y* = 0.91, *Q* ^2^ = 0.68. In these models, the predicted borderline cases are represented by filled circles (c, d) and the predicted low Silverberg score (grade I-II) serous carcinomas by open circles (d).

The 1D ^1^H CPMG HRMAS spectra are characterized by a high resolution and a low level of lipid signals which allowed the identification of a total of 38 different metabolites (Table 1). Typical 1D HRMAS spectra of healthy ovarian tissue and of the three different histological types of epithelial ovarian carcinoma tissues are presented in Figure 1 along with a partial metabolite assignment. Only the 4.7–0.5 ppm region used in the subsequent statistical analysis is shown. The PLS-DA analysis applied to the all-ovarian biopsies generated a two component PLS-DA model characterized by a faithful representation of the *Y* data (*R* ^2^ *Y* = 0.75) and by a good cumulative confidence criterion of prediction (*Q* ^2^ = 0.50). These results demonstrate an effective difference in the metabolic pattern of healthy tissues and the three histological types (Figure 2(a)). 

### 3.1. Endometrioid Carcinoma

A PLS-DA analysis performed on the same spectrum range 4.7–0.5 ppm revealed a clear separation between the 4 endometrioid carcinoma samples and the 3 controls (Figure 2(b), 2 component model, *R* ^2^ *Y* = 0.96, *Q* ^2^ = 0.45). Endometrioid cancer tissues showed a statistically significant higher level of total choline compounds (glycerophosphocholine, phosphocholine, and choline) and succinate. Healthy ovarian tissues are characterized by a higher level of aspartate. A PCA using only these discriminating metabolites confirmed a clear differentiation between endometrioid ovarian cancer and normal tissue (2 component model, *R* ^2^ *X* = 0.96 and *Q* ^2^ = 0.64). 

### 3.2. Mucinous Carcinoma

PLS-DA analysis performed using the spectrum range 4.7–0.5 ppm on the 6 mucinous carcinoma samples and the 3 controls also allowed a good separation (Figure 2(c), 2 component model, *R* ^2^ *Y* = 0.94, *Q* ^2^ = 0.69). Mucinous cancer tissues showed a statistically significant higher level of N-acetyl-lysine. The healthy ovarian tissue is characterized by a high level of taurine and myo-inositol. The PCA analysis based on the most discriminating metabolites showed a good separation between mucinous carcinoma and normal tissue (2 component model, *R* ^2^ *X* = 0.82, *Q* ^2^ = 0.69). The model was used to predict the two borderline mucinous cases: one specimen was classified with mucinous carcinoma samples, while the second was classified with control samples.

### 3.3. Serous Carcinoma

A PLS-DA analysis performed on the CPMG data, using the spectrum range 4.7–0.5 ppm, was not able to statistically separate serous carcinoma biopsies from control cases. Similarly, a PLS-DA based on the FIGO classification was not able to distinguish stage I, II, or III.

On the other hand, PLS-DA analysis based to the Silverberg grading allowed the separation of the higher grades of serous carcinoma (grade III, nine cases) from control tissues (Figure 2(d), 2 component model, *R* ^2^ *Y* = 0.91, *Q* ^2^ = 0.68). Serous cancer tissues showed a statistically significant higher level of acetate, N-acetyl-aspartate, alanine, lysine, threonine, glutamate, and succinate. The healthy ovarian tissues are characterized by a higher level of taurine and *β*-glucose. A PCA analysis based on these discriminating metabolites confirmed a clear separation between Silverberg grade III serous cancer and normal tissues (2 component model, *R* ^2^ *X* = 0.76, *Q* ^2^ = 0.6). 

The PLS-DA model obtained was also used to predict the classification of low Silverberg scores (grade I and II, 13 cases) and the borderline cases (3 cases). Figure 2(d) shows clearly that unlike the higher Silverberg score, it was not possible to find metabolically homogeneous group of low Silverberg score of serous cancer cases. On the other hand, this statistical model classified the 3 borderline tumors near the control tissues. 

### 3.4. Correlation of Spectroscopic Findings with Clinical Followup

**Figure 3:**
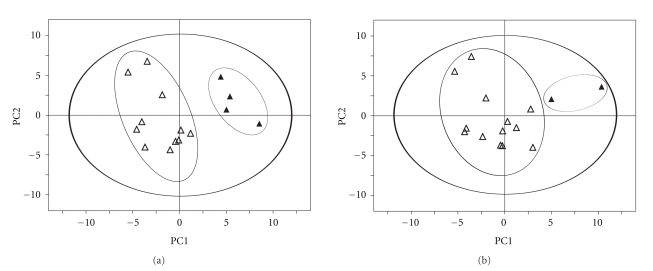
Score plot of the first two principal components (PC1 and PC2) from PLS-DA models obtained when correlating the metabolic data with: (a) Patient 24 months survival rate (superior to 24 months as open triangle inferior to 24 months as full triangle). Model parameters: *R* ^2^ *Y* = 0.85, *Q* ^2^ = 0.51. (b) Response to chemotherapy (positive response as open triangle negative response as full triangle). Model parameters: *R* ^2^ *Y* = 0.85, *Q* ^2^ = 0.42.

A PLS-DA analysis based on the 2-year survival rate, showed a clear separation between the 2 groups (Figure 3(a), 2 component model, *R* ^2^ *Y* = 0.85, *Q* ^2^ = 0.51) Patients presenting a better survival rate (*n* = 11) showed a higher level of glutamate, glutamine, aspartate, creatine, and glycine comparatively to the poor survival rate group (*n* = 4), which was characterized by a higher level of valine, leucine, and lysine. 

According to the response to chemotherapy, PLS-DA showed also a good separation between the group responding to treatment (*n* = 13) and the group resisting (*n* = 2) (Figure 3(b), 2 components, *R* ^2^ *Y* = 0.85, *Q* ^2^ = 0.42). Patients resisting treatment showed a higher level of succinate and 3-hydroxybutyrate, while the group responding to chemotherapy showed a higher amount of glutamate, glutamine, aspartate, and creatine. 

## 4. Discussion

The results presented in this study demonstrate clearly that the 3 histological types of epithelial ovarian carcinomas and normal tissue can be separated by the difference in their metabolic pattern. We observed that N-acetyl-aspartate (at 2.02 ppm) appeared to be a specific metabolic signature for serous carcinoma, while N-acetyl-lysine (at 2.04 ppm) is a potential signature for mucinous carcinoma. In the endometrioid carcinoma cases and in the normal ovarian tissues, we did not observe either N-acetyl-aspartate or N-acetyl-lysine. Only this small fraction of the NMR spectrum (2.02 to 2.04 ppm) allows to discriminate between these 3 histological types of ovarian cancer. The proximity of these two resonance and the –CH_3_ moiety of sialic acid or N-acetyl groups of glycoproteins (2.06 to 2.1 ppm) may potentially lead to some confusion *in vivo* ^1^H-MR spectroscopy [9, 11, 13, 14] or non-HRMAS^5^ *ex vivo* NMR studies of ovarian carcinoma cases. N-acetyl-aspartate is exclusively synthesized at high concentrations in the cytoplasm of neurons [21] and was detected in a few other tissues, as well as in peritoneal mast cells [22], retina, and lens [23] of the eye. The role of N-acetyl-aspartate in the solid part or in cyst fluid [8] of ovarian serous carcinomas and the other N-acetylated molecules resonating between 2.04 and 2.07 ppm in other cancers [9, 24–27] remains obscure. 

*β*-glucose was absent in all cases of serous cancer tissues. This observation is in accordance with many other cancer tissues studies [17, 28–30]. The absence of *β*-glucose is explained by the high-energy expenditure in cancer cells and elevated glucose turnover. This result was correlated with FDG-PET imaging studies, which demonstrated the sensitivity of FDG fixation in serous type of ovarian carcinomas, while endometrioid and mucinous types were sources of false negative results [3, 31–36]. In agreement with the literature, we also observed high levels of alanine and lactate in serous carcinoma indicating an impairment of the aerobic pathway, as well as elevated levels of glutamate, threonine (the principal amino acid of mucin), and lysine due to increased protein synthesis [8, 37]. In contrast to many other cancers [17, 28, 38, 39] and in agreement with gastric [37] or prostate cancers [37], the level of taurine was decreased in serous and mucinous ovarian carcinomas. This taurine deficiency may be associated with the loss of its protective role in cells, specifically in membrane stabilization as well as antioxidation and detoxification activities. 

An increase of the total choline-containing compounds (e.g., glycerophosphocholine, phosphocholine, and choline) has been reported as the most common feature in a large variety of tumors demonstrating biosynthetic and/or catabolic phosphatidylcholine-cycle pathways of cell membrane turnover [40, 41] and was proposed as fingerprints of tumor progression and/or as endpoints of therapeutic treatment [7, 12–14, 42]. Many authors have indeed concluded that the total choline-containing compounds could not be used as an indicator of malignancy but only as an indicator of proliferation of tumor cells (including in ovarian tumors) [4, 8, 14, 15, 43, 44]. In our case, we observed a significant higher level of total choline-containing compounds in all ovarian carcinomas, the free choline being more pronounced in endometrial carcinomas and phosphocholine/glycerophosphocholine in mucinous and serous carcinomas (Figure 1). 

A secondary aim of this study was to try to understand the biological potential and outcome of borderline tumors in order to adapt and avoid overtreatment, which is important, since these tumors occur in young females and represent 15% of epithelial ovarian cancer [4]. The statistical models generated in this study were able to predict borderline tumors, which present an intermediate metabolic pattern near the normal ovarian tissue, reinforcing the hypothesis of the existence of transitional metabolism during the progression of tumors [5]. The same transitional metabolism was also observed in low Silverberg score serous carcinoma cases which represent a metabolically heterogeneous group, intermediate between high Silverberg score serous carcinomas and normal ovarian tissue (Figure 2(d)). These results require confirmation with a larger number of cases.

Finally, to our knowledge, this paper presents the first retrospective study correlating metabolomics findings with clinical followup in serous ovarian cancers. PLS-DA analysis was able to predict survival by separating convincingly the group of patients with improved or inferior survival rates at 24 months. The statistical model was also able to predict tumors responsive or resistant to chemotherapy. These results may point to the presence of a different cell metabolism resulting in intrinsic drug resistance, noted in 30% of untreated tumors [45]. This complementary information may provide the opportunity to adapt the chemotherapeutic regimen for this heterogeneous group. Additional investigations are needed to elucidate the mechanisms of the intrinsic resistance in an attempt to identify possible panels of novel biomarkers and/or targets for therapeutic intervention. Despite the low number of tissue samples, our work based on metabolomics analysis of intact tissues using a nondestructive, rapid analysis protocol (30 min) provides a promising technique which may be applicable to the therapeutic management of patients [46]. It will clearly be necessary to conduct a multicenter medical study on a larger scale in order to confirm these preliminary very encouraging results. 

##  Conflict of Interests 

The authors declare that there is no conflict of interests. 

## Figures and Tables

**Table 1:** ^1^H resonance assignments of the metabolites present in cancerous and healthy human ovarian tissues.

†	Metabolites	Group	^1^H chemical shift (ppm)	^13^C chemical shift
1	Isoleucine	*δ*CH_3_	0.94	13.90
		*γ*CH_3_	1.01	17.29
		*γ*CH_2_	1.51	27.30
		*α*CH	3.65	62.34

2	Leucine	*δ*CH_3_	0.96	23.50
		*δ*′CH_3_	0.90	25.10
		*γ*CH	1.71	—
		*β*CH_2_	1.71	42.51
		*α*CH	3.74	56.05

3	Valine	*γ*CH_3_	0.99	19.26
		*γ*′CH_3_	1.05	20.70
		*β*CH	2.31	31.99

4	Ethanol	CH_3_	1.18	19.55
		CH2OH	3.66	60.11

5	Fatty acids (a)	(2)CH_2_	1.29	34.50
		(1)CH_2_	1.30	25.47

6	Fatty acids (b)	(2)CH_2_	2.02	27.22
		CH_2_	2.81	28.14
		(1)CH	5.32	130.55
		(2)CH	5.33	132.36

7	Fatty acids (a) (b)	(*n*)CH_2_	1.29	32.36

8	Fatty acids (c)	(2)CH_2_	1.56	27.20

9	Lactate	CH_2_	1.33	22.70
		CH	4.12	71.17

10	Lysine	*γ*CH_2_	1.44	24.66
		*δ*CH_2_	1.71	29.16
		*β*CH_2_	1.91	32.61
		*ε*CH_2_	3.01	41.92

11	Alanine	*β*CH_2_	1.48	18.89
		*α*CH	3.78	53.27

12	Glutamate	*β*CH_2_	2.07	29.77
		*γ*CH_2_	2.35	36.00
		*α*CH	3.77	57.15

13	Methionine	*ε*CH_2_	2.11	16.77

14	Glutamine	*β*CH_2_	2.14	—
		*γ*CH_2_	2.45	33.51
		*α*CH_2_	3.77	—

15	Aspartic acid	*β*CH_2_ (u)	2.70	39.17
		*β*CH_2_ (d)	2.80	39.17
		*α*CH	3.90	54.93

16	Choline	−N^+^-(CH_3_)_3_	3.21	—
		*β*CH_2_	3.52	69.96
		*α*CH	4.07	58.36

17	Phosphorylcholine	−N^+^-(CH_3_)_3_	3.23	56.58
		*β*CH_2_	3.62	68.89
		*α*CH	4.19	60.92

18	Glycerophosphocholine	−CH_2_-NH_2_^+^	3.24	—
		*α*CH_2_	4.33	62.03
		*β*CH_2_	3.69	68.62
		CH_2_-HPO_2_ (d)	3.88	69.37
		CH_2_OH	3.93	73.43
		CH_2_-HPO_2_ (u)	3.95	69.37

19	Arginine	*γ*CH_2_	1.72	26.67
		*β*CH_2_	1.92	30.26
		*δ*CH_2_	3.23	43.27

20	Taurine	−CH_2_-NH_2_^+^	3.26	50.22
		−CH_2_-SO_3_^−^	3.42	38.17

21	Proline	*δ*CH_2_ (u)	3.33	48.78
		*δ*CH_2_ (d)	3.41	48.78
		*α*CH	4.10	64.39

22	scyllo-Inositol	all Hs	3.34	76.37

23	myo-Inositol	C5H	3.27	77.11
		C1H, C3H	3.53	73.84
		C4H, C6H	3.61	75.06
		C2H	4.06	74.93

24	Glycine	*α*CH	3.56	44.17

25	Threonine	*α*CH	3.59	63.23
		*β*CH	4.26	68.81

26	Glycerol	1,3 CH_2_OH(u)	3.58	65.06
		1,3 CH_2_OH(d)	3.65	65.06
		−CH(OH)-	3.78	74.85

27	*β*-Glucose	C4H	3.41	72.44
		C3H, C5H	3.47	78.60
		C6H(u)	3.73	63.50
		C6H(d)	3.90	63.50
		C1H	4.65	—

28	*α*-Glucose	C1H	5.23	—

29	Serine	*α*CH	3.84	59.12
		*β*CH	3.99	63.09

30	Creatine	CH_3_	3.03	39.66
		CH_2_	3.94	56.44

31	Asparagine	*α*CH	4.00	54.15

32	Ascorbic acid	CH_2_OH	4.02	72.12
		C4H	4.52	80.96

33	Succinic acid	(*α*,*β*,CH_2_)	2.41	—

34	Glutathione	CH_2_-CONH	2.55	33.98
		CH_2_-SH	2.96	28.40
		CH-NH_2_	3.78	46.07
		CH-NH	4.58	58.40

35	Acetate	CH_2_	1.93	26.00

36	3-hydroxybutyrate	CH_2_	1.20	24.29
		*β*CH_2_(u)	2.29	49.11
		*β*CH_2_(d)	2.39	49.11
		CHOH	4.15	—

37	N Acetyl -Lysine	*β*CH_2_(u)	1.69	—
		*β*CH_2_(d)	1.80	—
		CH_2_	2.04	24.80
		*ε*CH_2_	3.00	—
		*α*CH	4.15	—

38	N Acetyl Aspartate	CH_2_	2.02	24.65
		*α*CH	4.39	55.97
